# Diagnostic Radiation Exposure in Patients with Inflammatory Bowel Disease

**DOI:** 10.1155/2019/2030735

**Published:** 2019-06-11

**Authors:** Catherine Langevin, Lysanne Normandeau, Mickael Bouin

**Affiliations:** ^1^Gastroenterology Division, Centre Hospitalier de l'Université de Montréal, Quebec, Canada; ^2^Health Physicist, Centre Hospitalier de l'Université de Montréal, Canada

## Abstract

**Background:**

Because of the chronic and relapsing nature of inflammatory bowel disease (IBD), which often requires characterization with CT scan, IBD patients might be exposed to a large amount of radiation. As a cumulative effective dose (CED) ≥ 100 mSv is considered significant for stochastic risks of cancer, it is important to monitor and control the radiation exposure of the IBD patients. In the present work, we aimed to quantify the mean CED in IBD patients to assess any harmful effects of radiation.

**Methods:**

This study includes 200 IBD patients, identified retrospectively, from the outpatient clinics of the Centre Hospitalier de l'Université de Montréal between January 1, 2010, and February 15, 2017, from the gastroenterologists' patients lists. The number and type of each radiology test performed were listed for each patient during the study period and the CED was calculated using our institution's dose index when available and standardized tables.

**Results:**

Among the 200 IBD patients, 157 patients had Crohn's disease (CD), 41 had ulcerative colitis (UC), and 2 had indeterminate colitis. The mean CED for IBD patients was 23.1 ± 45.2 mSv during a mean follow-up duration of 4.3 years. CED was higher among patients with CD than with UC (27.5 ± 49.5 versus 6.8 ± 14.8 mSv; p<0.01). Six patients were exposed to a high CED (>100 mSv) and all had CD.

**Conclusion:**

While potentially harmful levels of radiation exposure are of concern in only a small number of patients, strategies to limit such exposure are encouraged when clinically appropriate.

## 1. Background

Inflammatory bowel disease (IBD) is a chronic condition that includes Crohn's disease (CD) and ulcerative colitis (UC). It has a relapsing-remitting course and numerous complications, including both gastrointestinal and extraintestinal complications. Because of this, in addition to the workup done at diagnosis, IBD patients might undergo a lifetime of repeated imaging studies to evaluate the extent of intestinal involvement, monitor disease activity, and diagnose complications. This exposure to medical radiation occurs in a young population with an already well-documented increased risk of cancer due to IBD and enough remaining years of life to develop one [1, 2].

This practice can result in a large cumulative dose of radiation, mainly due to the use of CT scans which often represent the imaging modality of choice. For example, an abdominopelvic CT scan provides an effective radiation dose of approximately 10 mSv, while natural radiation is about 1.8 mSv per year in Canada (2.4 mSv worldwide). Between 1982 and 2006, the annual individual exposure to medical radiation increased from 0.54 mSv to 3.0 mSv on average, an increase of nearly 600%, half of which is derived from CT scans [3]. CT scan use for patients with Crohn's disease in the emergency department increased from 47% to 78% of the visits between 2001 and 2009 while the admission rate (68% to 71%), intestinal perforations, obstructions or abscesses (29% to 30%), and the sum of urgent diagnoses remained stable [3, 4].

Measuring a cumulative effective dose (CED) of radiation derived from imaging studies is inherently difficult. An exam's effective dose varies depending on the radiologist's protocol, the type of radiation, and each body part's vulnerability to radiation. The long-term biological risks of CED are modified by gender and age at exposure. Contemporary literature regarding radiation-induced cancer risk is arising from observational studies involving environmental, occupational, and medical exposures to radiation [5]. Several epidemiological studies on the atomic bomb survivors in Japan also showed that radiation is associated with stochastic risks of cancer, meaning that cancer probability is proportional to the radiation dose but its severity is independent from the dose [2, 6]. A linear-no-threshold model used to be the paradigm, but according to the Health Physics Society (HPS) in its most recent position statement on radiation risk, no adverse health effects have been demonstrated with a radiation dose < 100 mSv, when administered in divided doses over several years, in addition to the natural background radiation [7]. Nevertheless, the most frequent threshold defined as a high dose in studies related to CED in IBD patients is 50 mSv, probably because of the previous interpretation of radiation-induced cancer studies and because the mean duration of CED studies varied between 2 and 11 years. Thus, a 50 mSv of radiation during the period of study (2 to 11 years) is a proportionally high dose, given that those patients might continue to receive regular diagnostic radiation in the following years and as some of those studies were done in a pediatric population [8]. Quantifying medical radiation efficiently and determining its risks are still an area under development. International Commission on Radiological Protection (ICRP) emphasizes that medical radiation should not be treated like environmental or occupational radiation because it has a direct benefit for the patient [9]. With the judicious use of a scan, radiation risks are outweighed by the benefits of diagnosing a condition or complication that requires prompt management. Because of all those findings and the scarcity of data about CED in IBD patients we find it necessary and important to quantify CED in IBD patients.

## 2. Material and Methods

### 2.1. Study Design and Population

This retrospective study was conducted at Centre Hospitalier de l'Université de Montréal (CHUM) which is a tertiary-care center in Montreal, Canada. It was approved by the Research Ethics Committee of CHUM. Patients were identified from the outpatient clinics using the gastroenterologists' lists for the period between January 1, 2010, and February 15, 2017, until 200 patients were included. For each patient, an individual follow-up interval was determined. It was defined as the period between his first and last IBD appointment at our center during the study period. Participants over 18 years old were eligible for inclusion in the study if they had a diagnosis of IBD made by a gastroenterologist over two visits, as defined by standard clinical, endoscopic, and histologic criteria. Patients with indeterminate colitis (doubt between CD and UC) were not excluded as long as they were considered to have IBD. Patients with less than one year of follow-up time, cancer in the last 5 years, or history of transplantation were excluded. The patients' demographic, clinical, and radiological information were collected from our center's local electronic database and the provincial database. The electronic provincial database includes all radiology tests done in other hospitals. This is the only part of this study that necessitated direct verbal consent as established by the Ethics Committee. When a patient was included, he received a letter and a call to explain the study and request consent to access his provincial database. If the provincial database was unavailable, the patient was not excluded but only the local database was used.

### 2.2. Medical Radiation Exposure

The number and type of each radiology test performed were listed for each patient during the patient's follow-up time. A CED was calculated for each patient using our institution's dose index when available and standardized tables, otherwise, mostly from Bushberg and Mettler as shown in Table 1 for most common diagnostic imaging tests [10–12]. A high dose of radiation was defined as ≥100 mSv as it is the threshold from which an increased risk of cancer is documented [7].

### 2.3. Statistical Analysis

Statistical analyses were performed by using Microsoft Excel for the different groups including IBD, CD, and UC. No independent statistics were done for the subgroup of patients with indeterminate colitis because of the low number of patients (n=2), but these patients were included in the IBD group. Values are shown as mean ± SD and percentages. Categorical variables were analyzed by using the Chi-square test, while continuous variables were analyzed by using the Mann–Whitney* U* test or Student's t-test as appropriate. A p value <0.05 was considered to indicate statistical significance.

## 3. Results

**Figure 1:**
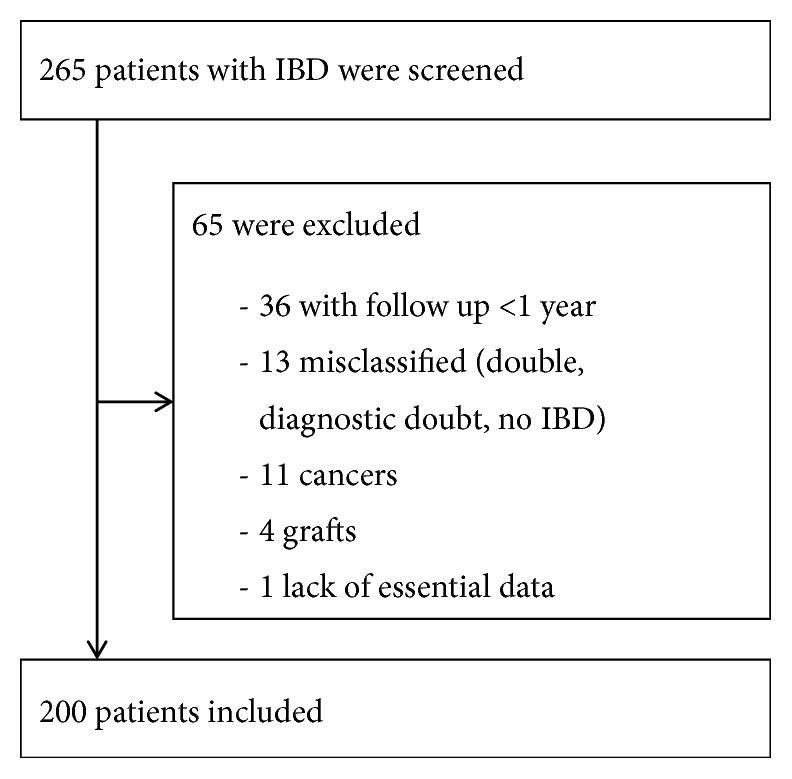
Study flowchart.

Among the 265 IBD patients identified, 65 patients were excluded (Figure 1). A total of 200 IBD patients were included, including 157 with Crohn's disease, 41 with ulcerative colitis, and 2 cases of indeterminate colitis. Baseline characteristics are shown in Table 2. Mean follow-up duration was 4.3 ± 1.8 years and varied between 1 and 7 years. During that time, 262 abdominal CT scans and 51 abdominal MRI were administered.

Mean cumulative effective dose (MCED) for IBD patients was 23.1 ± 45.2 mSv. Most of that radiation exposure was due to CT scans (19.72 ± 38.62 mSv). MCED was higher in CD patients than in UC patients (27.5 ± 49.5 versus 6.8 ± 14.8 mSv; p<0.01). During their follow-up, 51 (25.5%) IBD patients did not have even a single radiologic exam performed while 6 (3%) others were exposed to a high CED (≥ 100 mSv). These 6 IBD patients all had CD. They had between 5 and 25 scans each during the study period with a mean of 13.7 CT scans per patient. The difference of MCED between CD and UC remained significant when the analyses were repeated excluding these extreme cases.

The provincial database information was available for 163 IBD patients (81.5%) including 82.2% of all CD patients and 80.4% of all UC patients. Of those 163 patients, 111 (68.0%) had no radiologic exam outside of our hospital. The MCED derived from the provincial database was 2.45 ± 8.34 mSv and, therefore, 14.39% of the radiation dose calculated for each patient came from that database when it was available.

## 4. Discussion

This study quantified diagnostic radiation dose in IBD patients. Our results show that it takes 4 years for IBD patients to cumulate 23% of the radiation dose considered significant for stochastic risks of cancer. This exposure occurs in a young population at risk of cumulating additional diagnostic radiation over a lifetime and could be reduced by the judicious use of MRI, ultrasound, and biochemical markers. This is a very important finding, even if it largely underestimates reality, emphasizing the need for a radiation registry for IBD patients. Given that the mean age at diagnosis was 29 years in this study and the mean age at inclusion was 48 years, we suspect that many of the imaging studies done during those years, including the possibly more intensive workup done at diagnosis and during the first few years, are not captured in our present study.

A considerable effort was made to extensively detect all radiologic exams done during the study period. We extended our data collection from our local database to the provincial database in order to include all the exams done in other hospitals of our province. This register was available for 81.5% of patients. In a recent systematic review including 13 studies about radiation in IBD patients, 10 studies were found to use only their local database to list the imaging tests done while we showed that 14% of the CED for each patient could only be found in a centralized database [8]. Some exams done in private radiology clinics might have been missed, but this was not seen as a major loss as those private services are rarely used due to their exorbitant costs in an otherwise free healthcare system, suggesting that the quasi-totality of all imaging studies performed were accessible for our study. This small retrospective study has several limitations inherent to the difficulty to precisely quantify the radiation dose. Not every abdominal CT scan provides the same dose of radiation and there are significant variations between hospitals and within the same hospital according to the indication and context of the scan. Low-dose protocols are also becoming more common as well. This variability is representative of the reality. Our reasonable decision to use validated standardized tables to determine the effective radiation dose for each exam is based on the main accepted approach and improves external validity [8]. Assessing the specialty of the prescribing department (emergency, gastroenterology, other) would also have been relevant in order to target prevention interventions and improve quality of care.

Characterizing a group as heterogeneous as IBD patients also represented a challenge. For example, 51 (25.5%) patients did not have a single imaging study performed during their follow-up time while 6 (3%) patients were exposed to a high CED (≥ 100 mSv). In this study also CD patients consistently proved to be more exposed to medical radiation than UC patients, as in all other studies evaluating diagnostic radiation in IBD patients when a comparison was available [8]. Their predominance in this study is representative of the patients' lists that were provided but has an impact on the MCED for IBD patients. If our results were to be compared with a study that includes less CD patients, it would be preferable to use the respective results for CD patients and UC patients, rather than the composite radiation dose for all the IBD patients. Direct comparisons between studies can also be difficult because of the use of different end-points for describing diagnostic radiation such as a mean, a median, a number of mSv per year, a percentage of patients reaching a high CED (which could be defined as 50 or 100 mSv depending on the source), or the time required to reach such a dose. A standardized manner of expressing CED for IBD patients should be established.

Another consideration is that this study was performed in a tertiary-care center. It was considered an appropriate setting to study diagnostic radiation cumulation as multicomplicated patients are the ones at risk and these patients tend to end up in tertiary-care settings. Nevertheless, our main clientele remains to be patients who directly consulted our center and reflects typical IBD patients. The study population was a combination of patients from primary care setting and a few referrals. It included a broad range of patients and our results are concordant with contemporary literature.

While potentially harmful levels of radiation exposure is of concern in only a small number of IBD patients, strategies to limit such exposure are encouraged when clinically appropriate, especially among CD patients as they are significantly more at risk than UC patients. Resources should be invested to raise awareness about this issue and to successfully screen patients at risk of high diagnostic radiation exposure in order to modify our practice.

## Figures and Tables

**Table 1:** Mean radiation dose for common diagnostic radiologic tests.

Type of procedure	Effective dose (mSv)	Reference
Abdomen and pelvis scan	12.8	[10]
Virtual colonoscopy	10	[8]
Abdomen scan	8	[8]
Thorax scan	5.9	[10]
Barium enema	8	[8]
Upper gastrointestinal series	6	[8]
Small bowel series	5	[8]
Abdomen radiography	0.7	[8]
Chest radiography	0.02	[8]

mSv, millisievert.

**Table 2:** Patients and disease characteristics.

	IBD (n=200)	CD (n=157)	UC (n=41)	p-value
Patients' proportion	100%	78.50%	20.50%	

Mean age (years)	48.4 ± 13.9	48.5 ± 14.3	48.7 ± 12.5	0.92

Mean length of disease (years)	17.3 ± 11.6	19.1 ± 12.0	11.3 ± 7.3	<0.01

Mean length of follow up (years)	4.3 ± 1.8	4.4 ± 1.9	5.2 ± 5.9	0.86

Men, n (%)	77 (38.5%)	57 (36.3%)	17 (41.5%)	0.54

≥1 IBD-related intestinal resection, n (%)	69 (34.5%)	65 (41.4%)	3 (7.3%)	<0.01

Treatment, n (%)				

5-Aminosalicylate, n (%)	57 (28.5%)	27 (17.3%)	29 (70.7%)	<0.01

Immunomodulator, n (%)	59 (29.5%)	52 (33.1%)	7 (17.1%)	0.05

Biologic agent, n (%)	76 (38.0%)	70 (44.6%)	6 (14.6%)	<0.01

Chronic prednisone, n (%)	3 (1.5%)	3 (1.9%)	0 (0%)	0.86

Corticosteroids in the last year, n (%)	25 (12.5%)	22 (14.0%)	3 (7.3%)	0.25

Access to the provincial register, n (%)	163 (81.5%)	129 (82.2%)	33 (80.4%)	0.80

Mean CED (mSv)	23.1 ± 45.2	27.5 ± 49.5	6.8 ± 14.8	<0.01

Exposed to a CED ≥100 mSv, n (%)	6 (3%)	6 (3.8%)	0 (0%)	0.20

CD, Crohn's disease. CED, cumulative effective dose. IBD, intestinal bowel disease. mSv, millisievert. UC, ulcerative colitis.

*∗*p value between CD and UC.

## Data Availability

The data used to support the findings of this study is available from the corresponding author upon request.
